# Prediction of suitable regions of wild tomato provides insights on domesticated tomato cultivation in China

**DOI:** 10.1186/s12870-024-05410-z

**Published:** 2024-07-22

**Authors:** Ping Liu, Ruohan Xie, Guorong Xin, Yufei Sun, Shihao Su

**Affiliations:** School of Agriculture and Biotechnology, Sun Yat-sen University, Shenzhen, 518107 China

**Keywords:** Tomato, MaxEnt, *Solanum pimpinellifolium*, *Solanum lycopersicum*, Suitable region, Climate change

## Abstract

Climate change is one of the biggest challenges to the world at present. Tomato is also suffered from devastating yield loss due to climate change. The domesticated tomato (*Solanum lycopersicum*) is presumed to be originated from the wild tomato (*S. pimpinellifolium*). In this study, we compared the climate data of *S. pimpinellifollium* with the domesticated tomato, predicted the suitable regions of *S. pimpinellifollium* in China using MaxEnt model and assessed their tolerance to drought stress. We found that the predicted suitable regions of wild tomato are highly consistent with the current cultivated regions of domesticated tomato, suggesting that the habitat demand of domesticated tomato descended largely from its ancestor, hence the habitat information of wild tomato could provide a reference for tomato cultivation. We further predicted suitable regions of wild tomato in the future in China. Finally, we found that while average drought tolerance between wild and domesticated tomato accessions shows no difference, tolerance levels among wild tomato accessions exhibit higher variation, which could be used for future breeding to improve drought resistance. To summarize, our study shows that suitable regions of wild tomato provide insights into domesticated tomato cultivation in China.

**Supplementary Information:**

The online version contains supplementary material available at 10.1186/s12870-024-05410-z.

## Introduction

Agriculture is the most vulnerable sector to climate change, owing to its huge size and sensitivity to weather parameters, thereby causing huge economic impacts [1]. Climate change affects crop production through many different ways, including rising temperature, elevated CO_2_ concentration, and altered drought patterns [2, 3]. Crop wild relatives (CWRs) are reservoirs of valuable traits, including diverse forms of tolerance to both biotic and abiotic stresses, which remain crucial for the adaptation of modern cultivars to current and future climates. CWRs have been used for decades in crop improvement for enhancing plant performance [4]. Owing to their close evolutionary relationships with domesticated crops, traits from CWRs can be introgressed into domesticates with relative ease [5]. Domesticated tomato (*S. lycopersicum*) is an economically important crop that is also suffered from devastating yield loss due to global climate change and water deficiency [6]. Production and harvested area of domesticated tomato have consistently increased in the last decades in China and worldwide (FAO: https://www.fao.org/home/zh/; Fig. S1). China is the world’s largest tomato producer, and Xinjiang is the main production region in summer, while south China is the main tomato production region in winter seasons [7].

Wild tomato consists of 12 species of tomatoes (*Lycopersicon* group) including four species native to west South America, ranging from Ecuador to north Bolivia and Chile, with two endemic species in the Galápagos Islands [8]. Among the *Lycopersicon* group, *S. pimpinellifollium* is considered as a probable ancestor of domesticated tomato [9], favored by a very closed genomic relationship between them. *S. pimpinellifollium* is distributed from the south region of Ecuador to the north region of Chile [10]. The major climatic type of *S. pimpinellifollium* is arid and hot desert (Fig. S2, ArcGIS v10.8, ESRI, 2020) [10, 11]. In China, deserts are mainly distributed in the northwest part, including most areas of Xinjiang, central and north Gansu, and west Inner Mongolia. Previous work suggested that the most suitable regions for domesticated tomato in China are located along the Zhungeer basin, consistent with deserts in northwest China [12]. If biomes coincide between CWRs and domestic crops, it is expected that knowledge of the CWRs cultivation areas may provide insights for choosing cultivation areas for domesticated crops. The biome types between domesticated and wild tomatoes are similar, suggesting that the habitat of wild tomato may provide insights into domesticated tomato cultivation (Fig. S2).

Habitat Suitable Models (HSMs), such as MaxEnt, GARP, and CLIMEX, are widely used for predicting the potential suitable regions of various domestic crops or their CWRs in ratoon rice, wheat, and maize [13–16], as well as wild tomato and wild soybean [5, 17, 18]. Among these HSMs, the MaxEnt model is one of the most widely used models for predicting land suitability with high objectivity, accuracy, and geographical uniformity [19, 20]. In rice, the MaxEnt model was used to estimate potential paddy areas suitable only for ratoon rice but not for double-season rice [13]. In wild tomatoes, bioclimate envelopes of nine wild species based on the MaxEnt model were used to evaluate species divergence within the group [17]. In wild soybeans, potential distributions in past, present, and future periods were predicted using the MaxEnt model, supporting that climatic factors were highly responsible for eco-geographical differentiation [18].

To understand to predict future suitable cultivation areas for cultivated tomato under climate change scenarios in China, we used the MaxEnt model to predict the land suitability for *S. pimpinellifollium* based on different bioclimate variables in current and future periods. We also compared the climatic niche between domesticated tomato and *S. pimpinellifollium* quantitatively [19, 20], and found moderate niche overlap values between domesticated and wild tomatoes. We further evaluated drought tolerance levels between *S. pimpinellifollium* and domesticated tomato. The overall drought tolerance between wild and domesticated tomato accessions shows no difference, while tolerance levels among wild tomatoes exhibit higher variation, which could be used for future breeding to improve drought resistance. In conclusion, our study supports that suitable regions of CWRs provide a good reference for domesticated crop cultivation.

## Materials and methods

### Occurrence data

We compiled geo-referenced presence records by querying the Global Biodiversity Information Facility Data Portal (data.gbif.org; accessed 13/2/22). Sites that might be cultivated by humans or sites on the oceans were removed. The final occurrence dataset for *S. pimpinellifolium* contains 170 occurrence points (Table S1). The occurrence points of Xinjiang tomato and Winter tomato in China were collected from published articles and websites. A total of 29 and 45 occurrence points for Xinjiang tomato (Table S2) and Winter tomato (Table S3) were collected, respectively. Then, the latitude and longitude information of these occurrence points were acquired by Google Earth.

### Climatic data

Global Aridity Index data at 30 s (ca. 1 km at the equator) resolution were obtained from Aridity Index and Potential Evapo-Transpiration (ET0) Database v3 (https://www.plantplus.cn/en/dataset/1512D26417FF6A38; accessed 11/10/2022) [21]. Available water storage capacity data in mm/m of the soil unit at 30 s were downloaded from Harmonized World Soil Database v 1.2 (https://www.fao.org/soils-portal/soil-survey/soil-maps-and-databases/harmonized-world-soil-database-v12/en/; 1/5/2023) [22]. 19 bioclimatic layers (Table S4) from WorldClim version 2.0 (worldclim.org; accessed 19/12/2018) at 30 s resolution [23] were obtained from current or future periods (2021–2040, 2041–2060, 2061–2080, 2081–2100). RCP 4.5, was chosen as medium emission scenarios [24]. One widely used and high-resolution global circulation models (GCMs), HadGEM3 were used in these studies [25–28]. Climate data were extracted at each occurrence using ArcGIS v10.8 (ESRI, 2020).

### MaxEnt modeling

HSMs created by MaxEnt 3.4.4 k were applied to predict the suitable areas in geographic space. The geographically mapped results we used corresponded to the MaxEnt logistic output, which is best interpreted as a climatic suitability index for species over the landscape [29]. HSMs were separately calibrated with the native occurrence and background data of *S. pimpinellifolium*, which were projected onto China by using 19 bioclimatic layers and another two layer set. Variables reflecting the summer production season consist of bio 5, bio 8, bio 10, bio 13, bio 16 and bio 18. Those representing the winter production season include bio 6, bio 9, bio 11, bio 14, bio 17 and bio 19. MaxEnt was run using the 10,000 background points generated from the kernel density maps, with default settings, jackknifing, and logistic output [30]. The model was trained 10 times with cross validated replicated run type to verify the stability of the prediction accuracy [13]. An area under the curve (AUC) of the receiver operating characteristic plot is widely used as an indicator of model accuracy when using pseudo-absences, as with MaxEnt [31].

### Quantifying the niche dynamics

The extent of the study area has important effects on niche comparisons given its current distribution and the timescale considered in the study [20, 32, 33]. Ecuador and Peru administrative boundaries were used to define the range of *S. pimpinellifolium*, and, China administrative boundary was used to define the range of Xinjiang tomato and Winter tomato. Species data were projected onto the first two axes of a principal components analysis (PCA), depicting a multivariate climatic space calculated with the remaining climatic variables used in our study (Fig. S3). Following previous studies [20, 34], additional axes were not included since the first two explained a large proportion of the total climate variation (Fig. S3). The PCA was calibrated on climate factors distributed to both extents (referred to as PCAenv in [20]). Species occurrences were then transformed into species density using a kernel smoother in the gridded PCA environmental space (at a resolution of 100 * 100 cells) [20]. This approach reduces the risk that a difference between the numbers of two species records would cause an analytical bias in our results. This approach allowed species occupancy to be defined by correcting species densities by incorporating differences in environmental availability among tomato ranges [20].

The global overlap between the niches can be calculated using metrics such as Schoener’s D or Hellinger’s I, varying between 0 (no overlap) and 1 (total overlap) [19, 20]. We use the function ecospat.niche.overlap in the R package ecospat to calculate niche overlap [20, 33, 35]. Analyses were performed in R4.1.3 (RCoreTeam, 2022).

### Evaluation for seedling drought tolerance

Drought tolerance evaluation of tomato accessions was carried out in plant growth chambers during September–November, 2022. The tomato accessions were planted in plastic boxes (17 cm × 12 cm × 6.6 cm) filled with 0.25 kg soil at 25 °C in a 16‐h light/8‐h dark photoperiod. Twenty seeds per accession were sown in a culture dish with wet filter paper, and after germination, twelve healthy plants of each accession were transplanted into a box. Plants were watered every four days to keep the soil moist at the seedling stage. Watering was stopped at 30 days after germination for 10 days before rehydration. Recovery levels of seedlings (Table 1) were investigated three days after rehydration.

**Table 1:** Recovery levels of seedlings

ni	Recovery phenotypes
0	Fully recovered, or only leaf tips are slightly withered
1	Recovered, with no more than three dead leaves
2	At least one leaf recovered
3	Not recovered while the shoot apical meristem is active
4	Death of plants

The Recovery index (RI) based on the recovery level was calculated through the formula: RI = $\frac{\sum (xini)}{4\text{N}}\times 100\%$, where xi refers to the number of drought-damaged seedlings at all levels, ni: drought damage value at all levels, N: total number of seedlings. 20 accessions of *S. pimpinellifolium* and domesticated tomato (Table S5) were used for drought tolerance evaluation.

## Results

### Environmental variables of *S. pimpinellifolium*

**Fig. 1:**
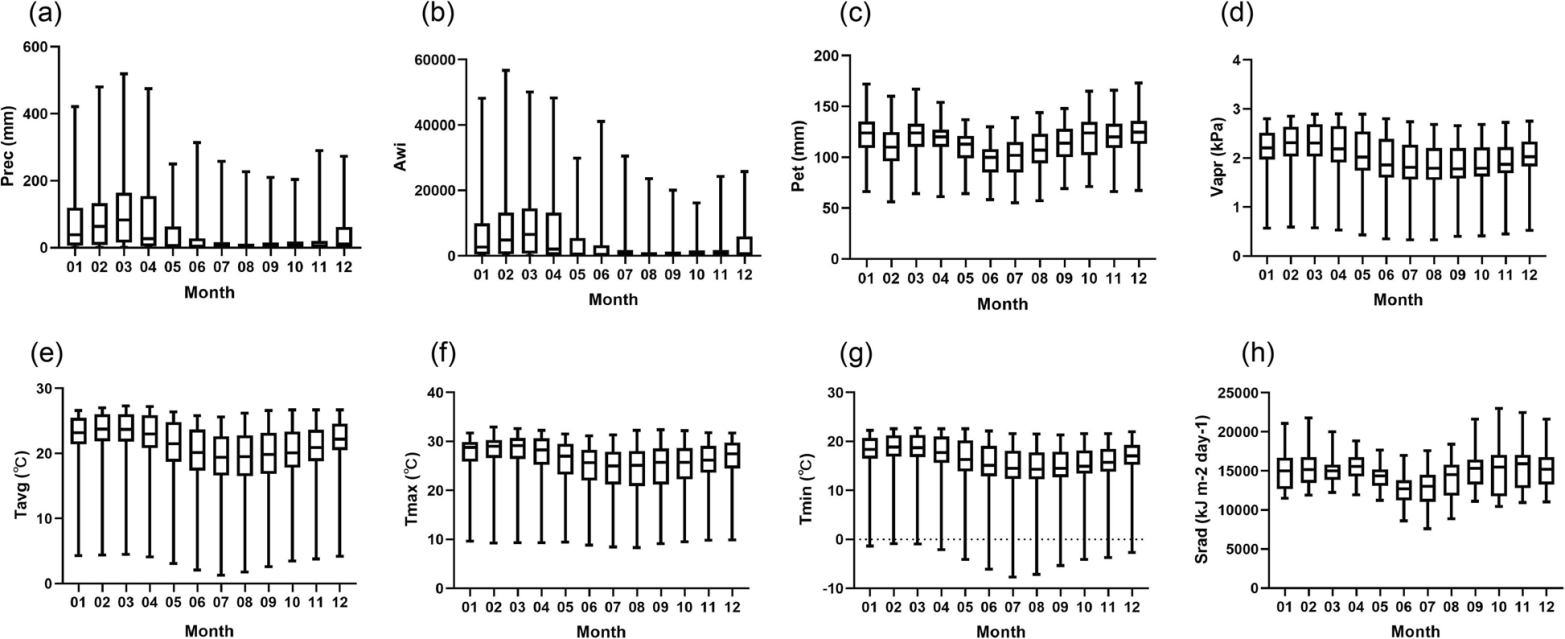
The monthly environmental variables of *S. pimpinellifolium*. **a** Precipitation (mm); **b** Aridity index (Awi); **c** Potential evapotranspiration (mm); **d** Water vapor pressure (kPa); **e** Average temperature (°C); **f** Maximum temperature (°C); **g** Minimum temperature (°C); **h** Solar radiation (kJ m-2 day-1)

The monthly environmental variables of *S.pimpinellifolium* were downloaded from WorldClim and HWSD websites and extracted by the R package. The environment of most sites from June to November is arid, derived from precipitation and aridity index (Fig. 1). The average monthly temperature of each month is between 15 and 25 ℃ at most sites.

**Fig. 2:**
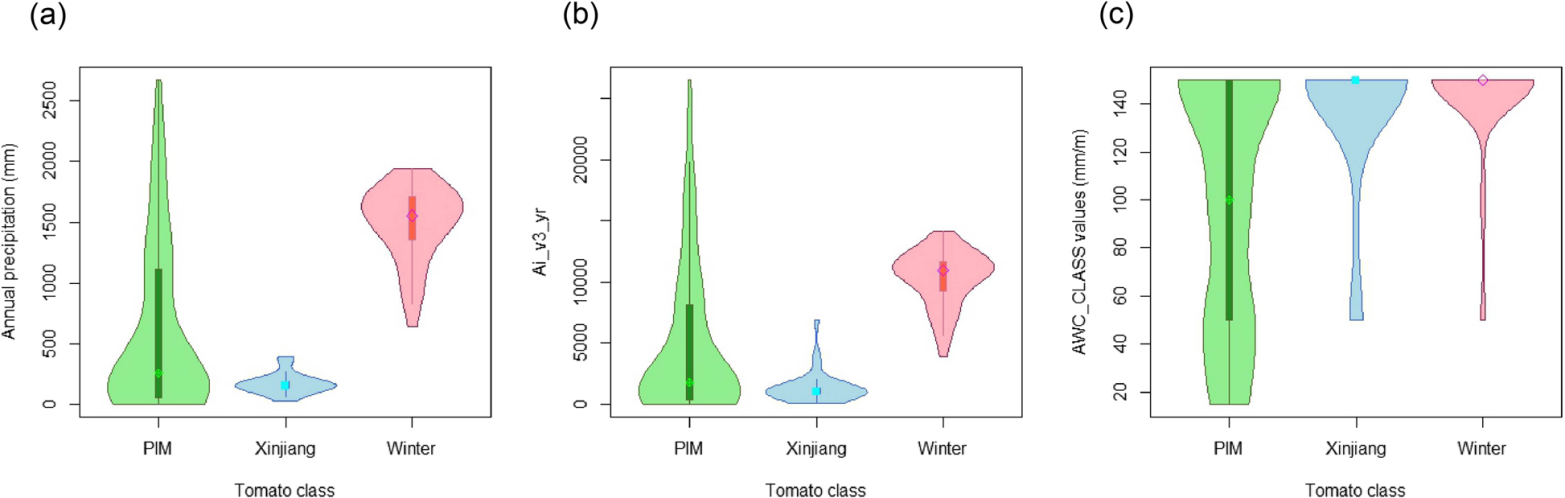
The parameter distribution related to precipitation in different groups of tomato. **a** Annual precipitation. **b** Annual aridity index. **c** Available water storage capacity in mm/m of the soil; PIM: *S. pimpinellifolium*, Xinjiang: Xinjiang tomato, Winter: Winter tomato

Among these variables, parameters related to precipitation include annual precipitation, aridity index, and available water storage capacity (Table S6-8). For *S. pimpinellifolium*, 43.90% of the sites locate in arid zones, 15.85% in semi-arid zones, 12.2% sites in semi-humid zones, 28.05% sites in humid zones (Table S6-8). We further compared the precipitation parameters of *S.pimpinellifolium* with those of cultivated tomatoes in China. For Xinjiang tomato, 82.76% of the sites are located in arid zones, while 17.24% sites in semi-arid zones (Table S6-8). For Winter tomato, 4.55% of the sites are located in semi-humid zones, and the rest sites are located in humid zones (Table S6-8). The precipitation condition of *S. pimpinellifolium* is similar to Xinjiang tomato, with higher diversity (Fig. 2a, b), suggesting that *S. pimpinellifolium* can survive in extremely dry soil. For Xinjiang tomato, the environment of most sites is arid or semi-arid, but the available water storage capacity is not low. For Winter tomato, the environment of most sites is humid and semi-humid, while the available water storage capacity is also similar (Fig. 2c). Although the annual precipitation for Winter tomato is high, precipitation in summer contributes to most of the annual precipitation, precipitation in tomato growth season is relatively low, supporting that arid and semi-arid regions are suitable for tomato cultivation.

### Relationship between land suitability of *S. pimpinellifolium* and environmental variables

AUC is a widely used tool for assessing the discriminatory capacity of models. The average AUC values across 10 runs are 0.897, 0.879, and 0.871 when using 19 bioclimatic variables, summer variables, and winter variables, respectively (Table S9), suggesting that the model provides a reliable prediction of the land suitability of *S. pimpinellifolium* in China.

The Annual Precipitation (bio 12), Precipitation of Driest Quarter (bio 17), Isothermality (BIO2/BIO7) (* 100) (bio 3), Precipitation of Driest Month (bio 14), Precipitation of Wettest Quarter (bio 16), Precipitation of Coldest Quarter (bio 19) have the highest effect on the modeling for *S. pimpinellifolium* within each category of environmental variables (Fig. S4). The highly suitable ranges for bio 12, bio 17, bio 3, bio 14, bio 16, and bio 19 are 6.34–572.53 mm, 0.76–10.29 mm, 35.1–76.93, 0.23–4.77 mm, 1.99–308.37 mm and 1.03–52.84 mm, respectively (Table S10). The most suitable values for bio 12, bio 17, bio 3, bio 14, bio 16, and bio 19 are 37.07 mm, 0.99 mm, 35.1, 0.23 mm, 33.11 mm, and 1.03 mm, respectively (Table S10). As shown in the jackknife figure (Fig. S4), except for bio 3, almost all the most important variables are related to precipitation*.*

### Potentially suitable regions of *S. pimpinellifolium* in China

**Fig. 3:**
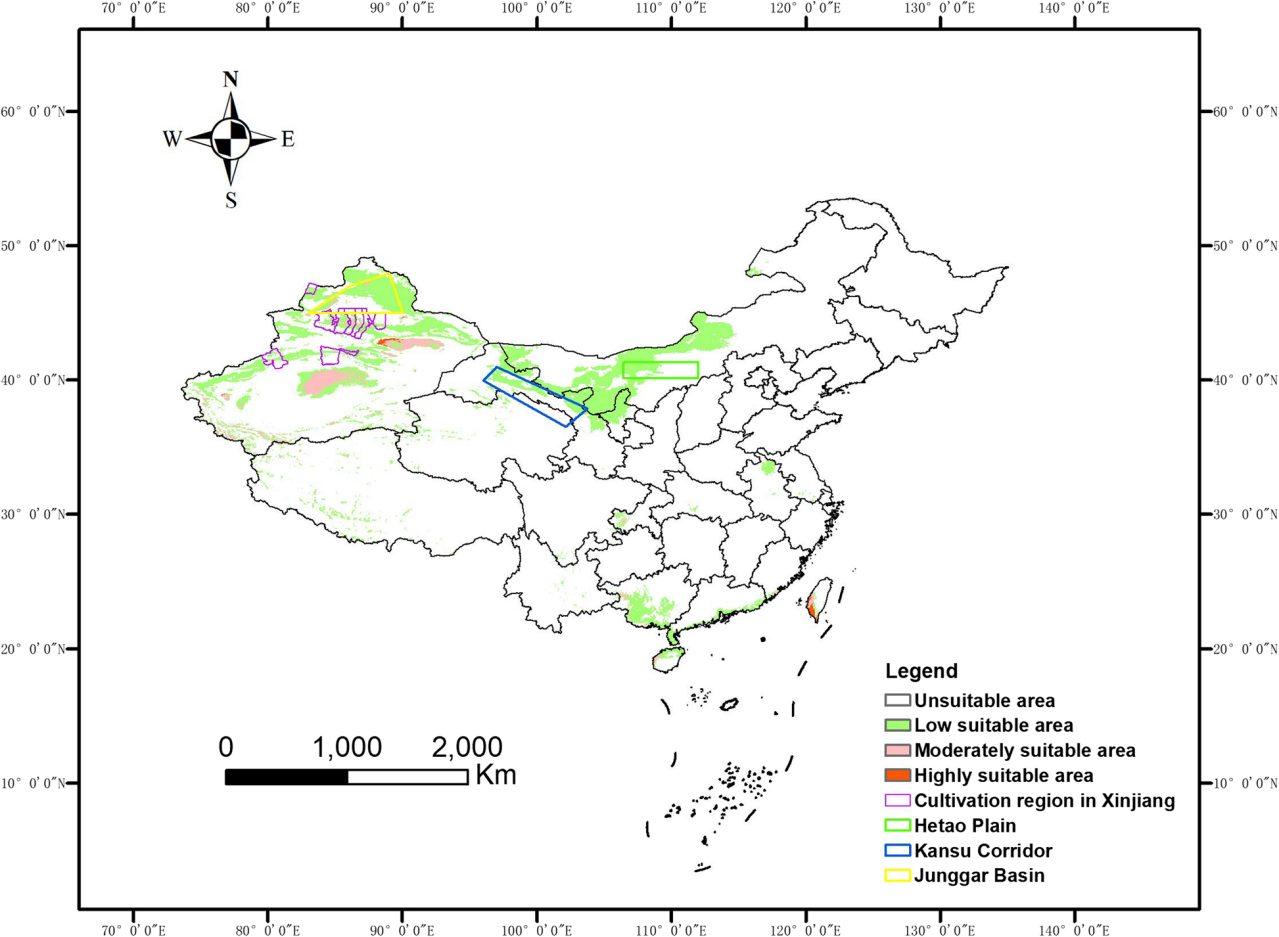
The predicted land suitability for *S. pimpinellifolium* in China by using 19 bioclimatic variables

The potentially highly suitable regions (the suitability value > 0.5) of *S. pimpinellifolium* in China by using 19 bioclimatic variables for modelling are located in small regions in north Xinjiang and south Taiwan island (Fig. 3). Moderately suitable regions (0.5 > the suitability value > 0.3) locate in part of Xinjiang, and low suitable regions (0.3 > the suitability value > 0.15) locate in large part of Xinjiang, north Gansu and west Inner Mongolia (Fig. 3). *S. pimpinellifolium* grows throughout the year in its native areas, while the domestic tomatoes are harvested within several months. Therefore, potentially suitable regions predicted by 19 bioclimatic variables may not be suitable for instructing domesticated tomato cultivation.

**Fig. 4:**
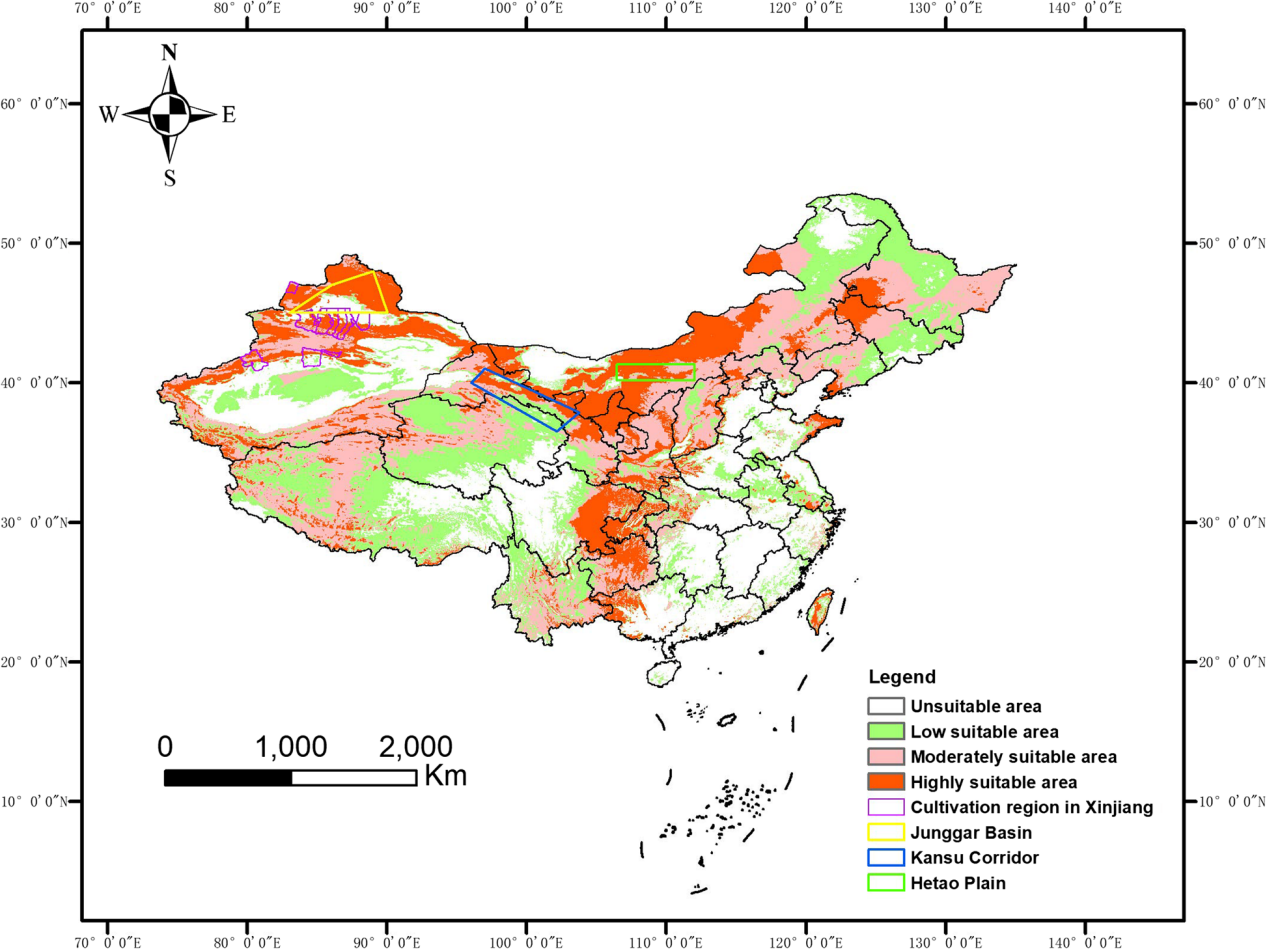
The predicted land suitability for *S. pimpinellifolium* in China by using summer variables (bio 5, bio 8, bio 10, bio 13, bio 16, bio 18)

We further re-predict the land suitability using separated summer (bio 5, bio 8, bio 10, bio 13, bio 16, bio 18) and winter variables (bio 6, bio 9, bio 11, bio 14, bio 17, bio 19). The potentially highly suitable regions of *S. pimpinellifolium* using summer variables, are located in a “V” shape region of China, including most of north and part of south Xinjiang, north Gansu, central and west Inner Mongolia, Ningxia, west Heilongjiang and Jilin, Liaoning and Shandong peninsulas, east Sichuan, Chongqing, north Guizhou, west Guangxi and central of Taiwan (Fig. 4). The highly suitable regions cover all the current tomato producing regions in Xinjiang.

**Fig. 5:**
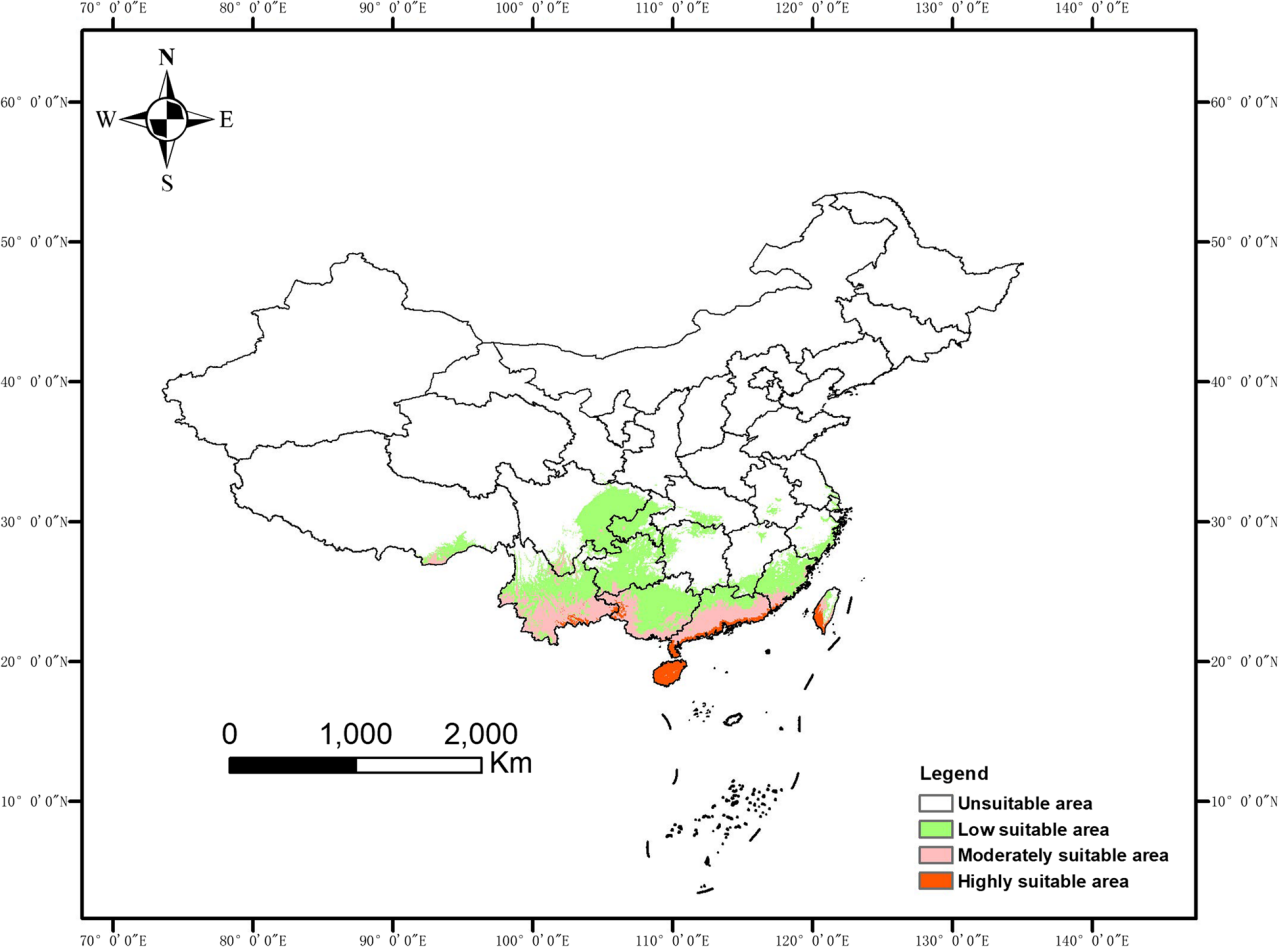
The predicted land suitability for *S. pimpinellifolium* in China by using winter variables (bio 6, bio 9, bio 11, bio 14, bio 17, bio 19)

The potentially highly suitable regions of *S. pimpinellifolium* using winter variables, are located in Hainan, Leizhou peninsula, and southwest Taiwan (Fig. 5). While moderately suitable regions contain south Yunnan and Guangxi, central and south Guangdong, and south Fujian. The predicted suitable regions are highly consistent with the current tomato cultivation regions in winter.

### Potential suitable regions of *S. pimpinellifolium* in China under future climate scenarios

**Fig. 6:**
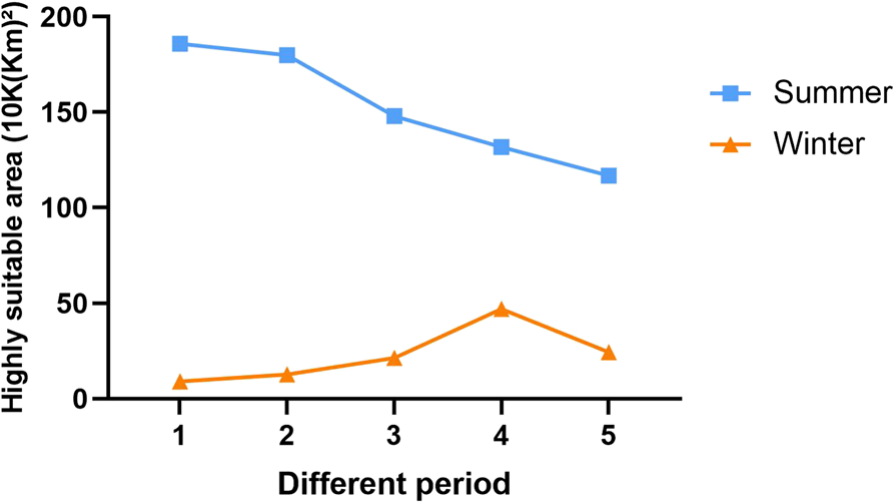
Area of highly suitable regions under climate change scenarios (10 K(km)^2^). 1–5 represent 1970–2000, 2020–2040, 2040–2060, 2060–2080 and 2080–2100, respectively

Compared with current situation, the overall highly suitable regions of *S. pimpinellifolium* in China predicted by summer variables will decline in the future (Fig. 6). Specifically, highly suitable regions in most parts of Xinjiang, Gansu, central and west Inner Mongolia, east Sichuan will decrease, while new regions in east Inner Mongolia, west Heilongjiang, west Jilin, west Liaoning and Shandong peninsulas are expected to emerge in the 2030s (Fig. S5). Summer highly suitable regions of *S. pimpinellifolium* in China keep declining until the 2100s (Fig. S5). In 2081–2100, there will be almost no highly suitable regions in northwest China, east Sichuan, and Shandong peninsula in the summer seasons (Fig. S5).

The potentially highly suitable regions of *S. pimpinellifolium* in China in the future modeled by winter variables will increase until the 2070s and then decline from the 2080s (Fig. 6). West Sichuan and east Chongqing are expected to become highly or moderately suitable regions from the 2020s (Fig. S6). South Guangdong and Guangxi will become moderately suitable regions in the future (Fig. S6). These results indicate that future tomato cultivation in China may shift from summer to winter seasons, from north to south regions.

### The niche overlap between domesticated tomato and *S. pimpinellifolium*

**Fig. 7:**
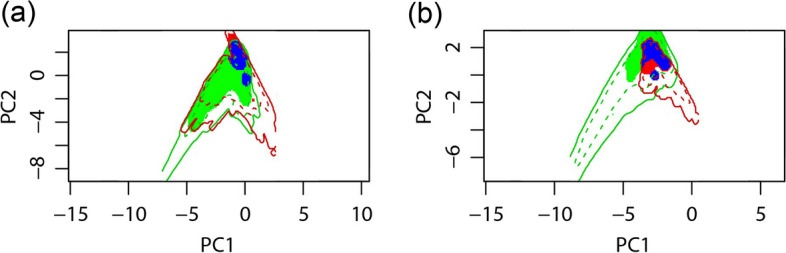
Climatic niche overlaps between domesticated tomato and *S. pimpinellifolium*. **a** Xinjiang tomato versus *S. pimpinellifolium*. **b** Winter tomato versus *S. pimpinellifolium*. Blue-colored areas indicate the niche overlaps between domesticated tomato and *S. pimpinellifolium*, green-colored areas indicate the niche of *S. pimpinellifolium*, red-colored areas indicate the niche of domesticated tomato, colored outlines indicate the background (or available) climate for each taxon

We further compared the climatic niche between domesticated tomato and *S. pimpinellifolium* by niche overlap (Fig. 7). The niche overlap values are, 0.41 (Schoener’s D value = 0.41, Hellinger’s I value = 0.62) between Xinjiang tomato and *S. pimpinellifolium*, 0.34 (Schoener’s D value = 0.34, Hellinger’s I value = 0.53) between Winter tomato and *S. pimpinellifolium*. These niche overlap values are not low (niches with low overlap (Schoener’s D value < 0.3)) [20], and a larger overlap is found between Xinjiang and *S. pimpinellifolium*. The climatic niches of domestic tomato are almost perfectly included in the climatic niche of *S. pimpinellifolium* (Fig. 7), supporting that prediction of suitable regions of *S. pimpinellifolium* can instruct domesticated tomato production in China.

### Evaluation of drought tolerance levels between *S. pimpinellifollium* and domesticated tomato

**Fig. 8:**
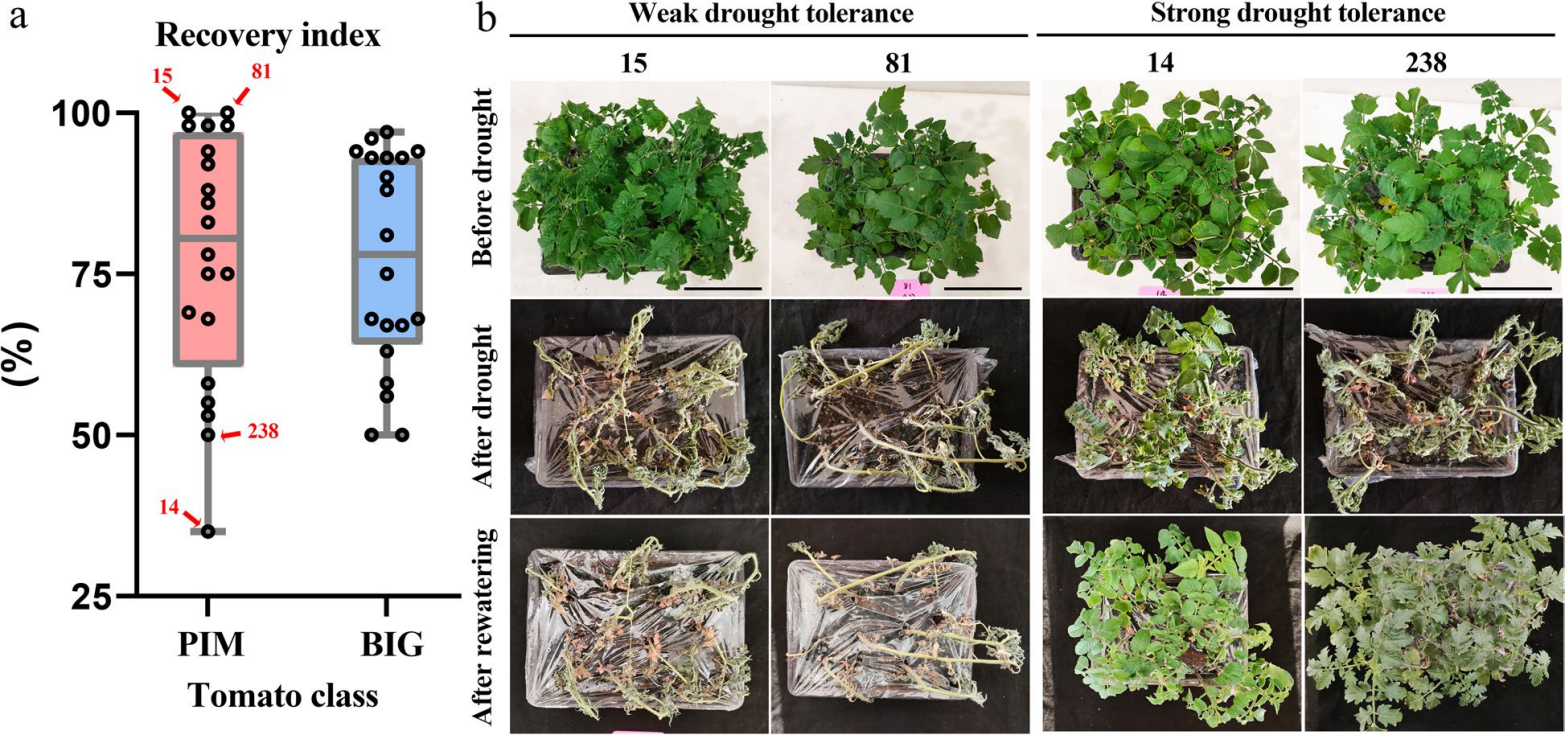
Evaluation of drought tolerance levels between *S. pimpinellifollium* and domesticated tomato. **a** RI values between different groups of tomato, PIM: *S. pimpinellifolium*, BIG: domesticated tomato. **b** Plant phenotypes of *S. pimpinellifolium*. 15 and 81 are the two strongest tolerant accessions, while 14 and 238 are the two weakest tolerant accessions

To investigate the drought responses of different accessions of tomato varieties to water deficit and recovery, the plant phenotypes were recorded at three stages: 1 day before the drought treatment; the last day of drought treatment, and 3 days after water recovery (Fig. 8, Fig. S7). We further calculated drought tolerance RI values between *S. pimpinellifollium* and domesticated tomato (Table S11). Consistent with their overlapped climatic niches, the average drought tolerance between wild and domesticated tomato accessions shows no significant difference (Fig. 8, S7, *p* = 0.495). However, tolerance levels among *S. pimpinellifolium* accessions exhibit higher variation, with both weak tolerant accessions such as accessions 15 and 81, as well as strong tolerant accessions including accessions 14 and 238 (Fig. 8b). All plants died after drought treatment and failed to recover in accessions 15 and 8; while all plants recovered with only several slightly withered leaf tips in accessions 14 and 238 (Fig. 8b). These wild tomato accessions could be used for future breeding to improve drought resistance of cultivated tomato.

## Discussion

In our study, we used the MaxEnt model to predict the suitable growing regions for *S. pimpinellifolium* in China. Accurate prediction is achieved partially due to the superlative analytical capacity of the MaxEnt model [29]. The MaxEnt model predicted the land suitability of *S. pimpinellifolium* under natural conditions is highly consistent with the actual cultivated regions of cultivated tomato in China. A previous study found that regions along the Zhungeer basin eastward to the Kansu corridor, and eastward to the Hetao plain are the most suitable regions for domesticated tomato [12]. These regions are included in our predicted regions of *S. pimpinellifolium* in summer China (Fig. 4). Besides these regions, we also found newly suitable regions, including southwest Heilongjiang, west Jilin, Liaoning peninsula, Shandong peninsula, east Sichuan, Chongqing, north Guizhou, west Guangxi, and part of Taiwan. For tomato cultivation in winter, our study suggests that Hainan, the Leizhou peninsula and southwest Taiwan are highly suitable for *S. pimpinellifolium* (Fig. 5).

Climate change increases the frequency of extreme weather such as droughts, floods, heatwaves, and extreme precipitation, and it affects agricultural productivity all over the world [2, 3, 36]. It is important to find the suitable regions for crops to adapt to climate change. Under climate change scenarios, suitable regions for *S. pimpinellifolium* will decline and move to the east in summer, while increasing and shifting to the north in winter in the future (Fig. S5-S6). It offers a reference to find suitable regions for domesticated tomato to deal with future climate change. To solve this, one alternative is to find out high temperature or drought-resistant varieties that grow in summer; another effective, cost-saving, and eco-friendly approach could be a gradual increase of tomato cultivation areas in winter China in the future.

One highlight of our study is that we performed physiological experiments to evaluate the drought tolerance between domesticated and wild tomatoes quantitatively. Leaf wilting under drought is a pivotal index of drought response [37], this is our major parameter to evaluate plant drought tolerance. Our study not only demonstrates that the average drought tolerance behaviors are similar between wild and domesticated tomato accessions, but also characterized potential wild tomato varieties with weaker or stronger drought resistance. These materials can be used to deal with climate change in the future. At the same time, however, our evaluation also suffers from limitations that should be addressed in future studies: first, drought resistance is a complicated trait, and therefore more parameters, such as plant height, leaf fresh/dry weight, stomatal conductance, root length should be assessed; second, plant numbers on each drought trial should be increased and randomized block design for statistical analysis should also be used. We are now working on them to address the mechanisms involved in drought response in tomato.

We predicted the potentially suitable regions for *S. pimpinellifolium* using the MaxEnt model for both current and future periods and compared the actual climatic niches as well as drought-resistant levels between *S. pimpinellifolium* and domesticated tomato (Fig. 8). However, this study does not consider other variables, such as cropping management factors that have an impact on plant cultivation. Furthermore, when evaluating the ecological niche overlap between *S. pimpinellifolium* and domesticated tomato, we only compared differences in drought-resistant levels. Other ecological factors, such as extreme temperatures and floods, should also be evaluated in further studies.

## Conclusions

Actual climatic data, habitat-suitable modeling, and physiological experiments favor that the climate and niche of *S. pimpinellifolium* and domesticated tomato are very similar. Hence, it is possible to use the suitable regions of *S. pimpinellifolium* to instruct domesticated tomato cultivation in China. This study represents a good illustration of finding suitable regions for crops based on the information of their CWRs under natural conditions. Our study can be a reference for governments to monitor their agricultural policies in the future.

### Supplementary Information

Supplementary Material 1.

Supplementary Material 2.

## Data Availability

All data generated or analyzed during this study are included in this published article and its supplementary information files.
