# Supplementary material for: Prediction of suitable regions of wild tomato provides insights on domesticated tomato cultivation in China
Source: BMC Plant Biol. 2024 Jul 22;24:693. doi: 10.1186/s12870-024-05410-z (PMC11265077; doi:10.1186/s12870-024-05410-z)
Supplement: Supplementary file 1 — Supplementary Material 1. [file 12870_2024_5410_MOESM1_ESM.docx]

Table S1. The occurrences of *Solanum pimpinellifolium.*

| **Scientific name** | **Longitude** | **Latitude** |
| --- | --- | --- |
| *Solanum pimpinellifolium* | -80.966 | -2.216 |
| *Solanum pimpinellifolium* | -80.92 | -2.25 |
| *Solanum pimpinellifolium* | -80.916667 | -2.25 |
| *Solanum pimpinellifolium* | -80.91666 | -2.25 |
| *Solanum pimpinellifolium* | -80.83333 | -1.03333 |
| *Solanum pimpinellifolium* | -80.83 | -1.03 |
| *Solanum pimpinellifolium* | -80.716667 | -4.883333 |
| *Solanum pimpinellifolium* | -80.683333 | -2.383333 |
| *Solanum pimpinellifolium* | -80.68333 | -2.38333 |
| *Solanum pimpinellifolium* | -80.68 | -2.38 |
| *Solanum pimpinellifolium* | -80.67 | -4.5 |
| *Solanum pimpinellifolium* | -80.65 | -1.07 |
| *Solanum pimpinellifolium* | -80.62 | -5.07 |
| *Solanum pimpinellifolium* | -80.5781 | -4.4167 |
| *Solanum pimpinellifolium* | -80.57 | -5.5 |
| *Solanum pimpinellifolium* | -80.5 | -2.35 |
| *Solanum pimpinellifolium* | -80.466667 | -3.566667 |
| *Solanum pimpinellifolium* | -80.433333 | -0.633333 |
| *Solanum pimpinellifolium* | -80.43333 | -0.63333 |
| *Solanum pimpinellifolium* | -80.43 | -0.63 |
| *Solanum pimpinellifolium* | -80.416667 | -0.6 |
| *Solanum pimpinellifolium* | -80.375 | -4.883333 |
| *Solanum pimpinellifolium* | -80.283333 | -2.35 |
| *Solanum pimpinellifolium* | -80.2833 | -2.35 |
| *Solanum pimpinellifolium* | -80.245 | -3.481389 |
| *Solanum pimpinellifolium* | -80.22 | -3.45 |
| *Solanum pimpinellifolium* | -80.2075 | -3.556111 |
| *Solanum pimpinellifolium* | -80.17 | -5.12 |
| *Solanum pimpinellifolium* | -80.116667 | -4.202778 |
| *Solanum pimpinellifolium* | -80.0844 | -5.2114 |
| *Solanum pimpinellifolium* | -80.0108 | -6.5344 |
| *Solanum pimpinellifolium* | -79.97 | -2.17 |
| *Solanum pimpinellifolium* | -79.9667 | -3.4583 |
| *Solanum pimpinellifolium* | -79.933333 | -1.866667 |
| *Solanum pimpinellifolium* | -79.933333 | -0.866667 |
| *Solanum pimpinellifolium* | -79.9333 | -0.86666 |
| *Solanum pimpinellifolium* | -79.916667 | -2.733333 |
| *Solanum pimpinellifolium* | -79.91666 | -0.4 |
| *Solanum pimpinellifolium* | -79.9166 | -2.73333 |
| *Solanum pimpinellifolium* | -79.9039 | -0.7444 |
| *Solanum pimpinellifolium* | -79.88333 | -3.5666 |
| *Solanum pimpinellifolium* | -79.85 | 0.866667 |
| *Solanum pimpinellifolium* | -79.768333 | 0.325 |
| *Solanum pimpinellifolium* | -79.76833 | 0.325 |
| *Solanum pimpinellifolium* | -79.73166 | -2.2225 |
| *Solanum pimpinellifolium* | -79.72 | -6.75 |
| *Solanum pimpinellifolium* | -79.72 | -2.1 |
| *Solanum pimpinellifolium* | -79.716667 | -6.75 |
| *Solanum pimpinellifolium* | -79.716667 | -6.15 |
| *Solanum pimpinellifolium* | -79.716667 | -2.1 |
| *Solanum pimpinellifolium* | -79.67 | -2.45 |
| *Solanum pimpinellifolium* | -79.666667 | -2.45 |
| *Solanum pimpinellifolium* | -79.65 | -1.06 |
| *Solanum pimpinellifolium* | -79.6144 | -2.6739 |
| *Solanum pimpinellifolium* | -79.567 | -7.4 |
| *Solanum pimpinellifolium* | -79.55 | -7.16667 |
| *Solanum pimpinellifolium* | -79.5242 | -6.402583 |
| *Solanum pimpinellifolium* | -79.517 | -1.817 |
| *Solanum pimpinellifolium* | -79.5 | -2.5 |
| *Solanum pimpinellifolium* | -79.483333 | -1.1 |
| *Solanum pimpinellifolium* | -79.4833 | -1.1 |
| *Solanum pimpinellifolium* | -79.467 | -1.583 |
| *Solanum pimpinellifolium* | -79.466667 | -7.45 |
| *Solanum pimpinellifolium* | -79.4553 | -1.7786 |
| *Solanum pimpinellifolium* | -79.3931 | -1.7133 |
| *Solanum pimpinellifolium* | -79.368056 | -0.575 |
| *Solanum pimpinellifolium* | -79.36666 | -0.58333 |
| *Solanum pimpinellifolium* | -79.334194 | -7.661389 |
| *Solanum pimpinellifolium* | -79.334167 | -7.661389 |
| *Solanum pimpinellifolium* | -79.236111 | -6.581389 |
| *Solanum pimpinellifolium* | -79.205833 | -6.6195 |
| *Solanum pimpinellifolium* | -79.203611 | -6.618333 |
| *Solanum pimpinellifolium* | -79.16194 | -7.83694 |
| *Solanum pimpinellifolium* | -79.15 | -0.25 |
| *Solanum pimpinellifolium* | -79.133333 | -7.833333 |
| *Solanum pimpinellifolium* | -79.1333 | -7.83333 |
| *Solanum pimpinellifolium* | -79.083 | -8.083 |
| *Solanum pimpinellifolium* | -79.05 | -7.2 |
| *Solanum pimpinellifolium* | -79.018333 | -7.671944 |
| *Solanum pimpinellifolium* | -79.009667 | -7.641917 |
| *Solanum pimpinellifolium* | -79.005833 | -7.638611 |
| *Solanum pimpinellifolium* | -79 | -6.3778 |
| *Solanum pimpinellifolium* | -78.97 | -8.17 |
| *Solanum pimpinellifolium* | -78.9667 | -8.1667 |
| *Solanum pimpinellifolium* | -78.958333 | -8.083333 |
| *Solanum pimpinellifolium* | -78.92 | -5.13 |
| *Solanum pimpinellifolium* | -78.9167 | -5.1333 |
| *Solanum pimpinellifolium* | -78.916667 | -5.133333 |
| *Solanum pimpinellifolium* | -78.91666 | -5.13333 |
| *Solanum pimpinellifolium* | -78.85 | -7.233333 |
| *Solanum pimpinellifolium* | -78.8 | -5.17 |
| *Solanum pimpinellifolium* | -78.8 | -5.16666 |
| *Solanum pimpinellifolium* | -78.77 | -5.28 |
| *Solanum pimpinellifolium* | -78.767071 | -1.427421 |
| *Solanum pimpinellifolium* | -78.766667 | -5.283333 |
| *Solanum pimpinellifolium* | -78.76666 | -5.28333 |
| *Solanum pimpinellifolium* | -78.69 | -7.24 |
| *Solanum pimpinellifolium* | -78.683333 | -7.983333 |
| *Solanum pimpinellifolium* | -78.6761 | -5.92 |
| *Solanum pimpinellifolium* | -78.672333 | -5.928 |
| *Solanum pimpinellifolium* | -78.666667 | -8.533333 |
| *Solanum pimpinellifolium* | -78.578897 | -0.187725 |
| *Solanum pimpinellifolium* | -78.567 | -8.05 |
| *Solanum pimpinellifolium* | -78.4778 | -8.1553 |
| *Solanum pimpinellifolium* | -78.331111 | -8.176389 |
| *Solanum pimpinellifolium* | -78.3 | -9.45 |
| *Solanum pimpinellifolium* | -78.283333 | -9.433333 |
| *Solanum pimpinellifolium* | -78.27 | -9.47 |
| *Solanum pimpinellifolium* | -78.233333 | -9.166667 |
| *Solanum pimpinellifolium* | -78.23 | -9.94 |
| *Solanum pimpinellifolium* | -78.21 | -9.94 |
| *Solanum pimpinellifolium* | -78.1869 | -1.4628 |
| *Solanum pimpinellifolium* | -78.167 | -10.067 |
| *Solanum pimpinellifolium* | -78.133333 | -9.008333 |
| *Solanum pimpinellifolium* | -78.04 | -10.28 |
| *Solanum pimpinellifolium* | -77.99 | -9.53 |
| *Solanum pimpinellifolium* | -77.9 | -9.541667 |
| *Solanum pimpinellifolium* | -77.865 | -9.55 |
| *Solanum pimpinellifolium* | -77.423917 | -10.605167 |
| *Solanum pimpinellifolium* | -77.423889 | -10.605278 |
| *Solanum pimpinellifolium* | -77.416667 | -11.1 |
| *Solanum pimpinellifolium* | -77.15 | -12.2 |
| *Solanum pimpinellifolium* | -77.108333 | -11.475 |
| *Solanum pimpinellifolium* | -77.03 | -12.15 |
| *Solanum pimpinellifolium* | -77.02 | -12.08 |
| *Solanum pimpinellifolium* | -77 | -11.533333 |
| *Solanum pimpinellifolium* | -77 | -11.6 |
| *Solanum pimpinellifolium* | -76.9833 | -12.0744 |
| *Solanum pimpinellifolium* | -76.95 | -12.083333 |
| *Solanum pimpinellifolium* | -76.943333 | -12.074722 |
| *Solanum pimpinellifolium* | -76.908056 | -11.6775 |
| *Solanum pimpinellifolium* | -76.865189 | -11.309994 |
| *Solanum pimpinellifolium* | -76.8333 | -7 |
| *Solanum pimpinellifolium* | -76.816667 | -12.133333 |
| *Solanum pimpinellifolium* | -76.8131 | -11.3647 |
| *Solanum pimpinellifolium* | -76.8053 | -12.135 |
| *Solanum pimpinellifolium* | -76.8 | -11.966667 |
| *Solanum pimpinellifolium* | -76.7667 | -11.9825 |
| *Solanum pimpinellifolium* | -76.733333 | -12.5 |
| *Solanum pimpinellifolium* | -76.716667 | -11.95 |
| *Solanum pimpinellifolium* | -76.716667 | -12.033333 |
| *Solanum pimpinellifolium* | -76.7 | -11.8 |
| *Solanum pimpinellifolium* | -76.678 | -11.9 |
| *Solanum pimpinellifolium* | -76.666667 | -11.666667 |
| *Solanum pimpinellifolium* | -76.66666 | -11.66666 |
| *Solanum pimpinellifolium* | -76.663333 | -11.900833 |
| *Solanum pimpinellifolium* | -76.65 | -11.858333 |
| *Solanum pimpinellifolium* | -76.516667 | -11.9 |
| *Solanum pimpinellifolium* | -76.5 | -12.766667 |
| *Solanum pimpinellifolium* | -76.4397 | -11.8847 |
| *Solanum pimpinellifolium* | -76.4 | -13.083333 |
| *Solanum pimpinellifolium* | -76.4 | -13.116667 |
| *Solanum pimpinellifolium* | -76.39 | -13.09 |
| *Solanum pimpinellifolium* | -76.13 | -12.97 |
| *Solanum pimpinellifolium* | -75.916667 | -13.733333 |
| *Solanum pimpinellifolium* | -75.4 | -10.5667 |
| *Solanum pimpinellifolium* | -75.144444 | -13.082222 |
| *Solanum pimpinellifolium* | -75.1444 | -13.0822 |
| *Solanum pimpinellifolium* | -75.058333 | -14.65 |
| *Solanum pimpinellifolium* | -75 | -10 |
| *Solanum pimpinellifolium* | -74.95 | -14.833333 |
| *Solanum pimpinellifolium* | -74.375627 | -9.163761 |
| *Solanum pimpinellifolium* | -74.14 | -9.1839 |
| *Solanum pimpinellifolium* | -73.25 | -16.4 |
| *Solanum pimpinellifolium* | -72.717 | -12.817 |
| *Solanum pimpinellifolium* | -72.716319 | -15.801586 |
| *Solanum pimpinellifolium* | -72.7 | -12.866667 |
| *Solanum pimpinellifolium* | -72.05 | -13 |
| *Solanum pimpinellifolium* | -71.57 | -12.47 |
| *Solanum pimpinellifolium* | -71.56666 | -12.46666 |

Table S2. The occurrences of Xinjiang tomato.

| **Scientific name** | **Longitude** | **Latitude** |
| --- | --- | --- |
| Xinjiang tomato | 87.56 | 43.82 |
| Xinjiang tomato | 87 | 44.22 |
| Xinjiang tomato | 88.35 | 44.09 |
| Xinjiang tomato | 86.61 | 44.24 |
| Xinjiang tomato | 86.07 | 44.4 |
| Xinjiang tomato | 90.29 | 44.45 |
| Xinjiang tomato | 90.05 | 42.45 |
| Xinjiang tomato | 85.02 | 44.58 |
| Xinjiang tomato | 83.17 | 46.82 |
| Xinjiang tomato | 84.26 | 44.43 |
| Xinjiang tomato | 84.28 | 46.6 |
| Xinjiang tomato | 89.68 | 43.67 |
| Xinjiang tomato | 82.86 | 45.94 |
| Xinjiang tomato | 84.61 | 41.82 |
| Xinjiang tomato | 85.99 | 42.09 |
| Xinjiang tomato | 85.01 | 42.83 |
| Xinjiang tomato | 87.49 | 42.11 |
| Xinjiang tomato | 86.88 | 41.9 |
| Xinjiang tomato | 80.47 | 41.61 |
| Xinjiang tomato | 79.22 | 41.29 |
| Xinjiang tomato | 77.04 | 38.25 |
| Xinjiang tomato | 87.54 | 44.3 |
| Xinjiang tomato | 86.04 | 44.29 |
| Xinjiang tomato | 85.76 | 43.99 |
| Xinjiang tomato | 87.54 | 43.91 |
| Xinjiang tomato | 87.01 | 44.01 |
| Xinjiang tomato | 87.07 | 44 |
| Xinjiang tomato | 84.91 | 44.42 |
| Xinjiang tomato | 86.03 | 44.3 |

Table S3. The occurrences of Winter tomato.

| **Scientific name** | **Longitude** | **Latitude** |
| --- | --- | --- |
| Winter tomato | 108.22 | 23.22 |
| Winter tomato | 108.9 | 23.18 |
| Winter tomato | 109.21 | 22.81 |
| Winter tomato | 108.06 | 22.93 |
| Winter tomato | 108.63 | 23.5 |
| Winter tomato | 106.76 | 23.8 |
| Winter tomato | 106.88 | 23.73 |
| Winter tomato | 107.17 | 23.63 |
| Winter tomato | 107.59 | 23.55 |
| Winter tomato | 106.53 | 23.92 |
| Winter tomato | 109.94 | 22.79 |
| Winter tomato | 110.16 | 22.68 |
| Winter tomato | 110.57 | 22.79 |
| Winter tomato | 109.13 | 22.25 |
| Winter tomato | 108.79 | 21.82 |
| Winter tomato | 108.57 | 22.18 |
| Winter tomato | 116.26 | 21.82 |
| Winter tomato | 114.42 | 23.09 |
| Winter tomato | 113.93 | 22.76 |
| Winter tomato | 110.69 | 21.35 |
| Winter tomato | 109.96 | 21.4 |
| Winter tomato | 111.16 | 21.66 |
| Winter tomato | 113.68 | 23 |
| Winter tomato | 113.05 | 23.55 |
| Winter tomato | 113.43 | 24.94 |
| Winter tomato | 101.85 | 25.75 |
| Winter tomato | 99.18 | 25.11 |
| Winter tomato | 103.51 | 23.29 |
| Winter tomato | 102.89 | 23.69 |
| Winter tomato | 100.63 | 25.87 |
| Winter tomato | 101.54 | 24.56 |
| Winter tomato | 103.16 | 26.98 |
| Winter tomato | 103.4 | 23.38 |
| Winter tomato | 110.32 | 20.01 |
| Winter tomato | 109.52 | 18.26 |
| Winter tomato | 110.89 | 19.78 |
| Winter tomato | 110.42 | 19.22 |
| Winter tomato | 110.34 | 19.47 |
| Winter tomato | 110 | 19.73 |
| Winter tomato | 109.7 | 19.81 |
| Winter tomato | 109.33 | 19.57 |
| Winter tomato | 108.85 | 19.04 |
| Winter tomato | 109.03 | 18.64 |
| Winter tomato | 110 | 18.54 |
| Winter tomato | 108.95 | 19.21 |

Table S4. 19 bioclimatic variables

| **name** | **mean** |
| --- | --- |
| BIO1 | Annual Mean Temperature |
| BIO2 | Mean Diurnal Range (Mean of monthly (max temp - min temp)) |
| BIO3 | Isothermality (BIO2/BIO7) (* 100) |
| BIO4 | Temperature Seasonality (standard deviation *100) |
| BIO5 | Max Temperature of Warmest Month |
| BIO6 | Min Temperature of Coldest Month |
| BIO7 | Temperature Annual Range (BIO5-BIO6) |
| BIO8 | Mean Temperature of Wettest Quarter |
| BIO9 | Mean Temperature of Driest Quarter |
| BIO10 | Mean Temperature of Warmest Quarter |
| BIO11 | Mean Temperature of Coldest Quarter |
| BIO12 | Annual Precipitation |
| BIO13 | Precipitation of Wettest Month |
| BIO14 | Precipitation of Driest Month |
| BIO15 | Precipitation Seasonality (Coefficient of Variation) |
| BIO16 | Precipitation of Wettest Quarter |
| BIO17 | Precipitation of Driest Quarter |
| BIO18 | Precipitation of Warmest Quarter |
| BIO19 | Precipitation of Coldest Quarter |

Table S5. Summary of different accessions of tomato varieties.

| **Number** | **Group** | **Name** | **Categories** | ***Botanical variety*** | **TGRC** |
| --- | --- | --- | --- | --- | --- |
| 14 | PIM |  | Wild species | *Solanum pimpinellifolium* | LA2093 |
| 15 | PIM |  | Wild species | *Solanum pimpinellifolium* | LA1246 |
| 18 | PIM |  | Wild species | *Solanum pimpinellifolium* | LA1579 |
| 20 | PIM | Sechin | Wild species | *Solanum pimpinellifolium* | LA0442 |
| 21 | PIM |  | Wild species | *Solanum pimpinellifolium* | LA1375 |
| 23 | PIM | Pisiquillo | Wild species | *Solanum pimpinellifolium* | LA1269 |
| 24 | PIM | EI Pinon, Asia | Wild species | *Solanum pimpinellifolium* | LA1521 |
| 25 | PIM |  | Wild species | *Solanum pimpinellifolium* | - |
| 67 | PIM | Atacames | Wild species | *Solanum pimpinellifolium* | LA1237 |
| 81 | PIM | Punto Cuatro | Wild species | *Solanum pimpinellifolium* | LA1582 |
| 127 | PIM | Tumbes south | Wild species | *Solanum pimpinellifolium* | LA1617 |
| 135 | PIM | Balsa Huaico | Wild species | *Solanum pimpinellifolium* | LA2181 |
| 164 | PIM | Corral Quemado | Wild species | *Solanum pimpinellifolium* | LA2183 |
| 238 | PIM | Santo Tome | Wild species | *Solanum pimpinellifolium* | LA1478 |
| 265 | PIM | Viru to Galumga | Wild species | *Solanum pimpinellifolium* | LA1589 |
| 277 | PIM |  | Wild species | *Solanum pimpinellifolium* | LA0480 |
| 278 | PIM |  | Wild species | *Solanum pimpinellifolium* | LA1242 |
| 279 | PIM |  | Wild species | *Solanum pimpinellifolium* | LA1341 |
| 280 | PIM |  | Wild species | *Solanum pimpinellifolium* | LA1596 |
| 281 | PIM |  | Wild species | *Solanum pimpinellifolium* | LA2184 |
| 289 | PIM | SAL1875 | Wild species | *Solanum pimpinellifolium* | LA1595 |
| 2 | BIG | Moneymaker | Vintage Fresh Market | *Solanum lycopersicum* | LA2706 |
| 3 | BIG | Hawaii 7998 | Inbreed line | *Solanum lycopersicum* | LA3856 |
| 4 | BIG | Edkawi | Vintage Fresh Market | *Solanum lycopersicum* | LA2711 |
| 9 | BIG | Ailsa Craig | Vintage Fresh Market | *Solanum lycopersicum* | LA2838A |
| 45 | BIG | Earliana | Vintage Processing | *Solanum lycopersicum* | LA3238 |
| 60 | BIG | Huachinango | Latin American cultivar | *Solanum lycopersicum* | LA1459 |
| 77 | BIG |  | Cocktail tomato | *Solanum lycopersicum* | - |
| 84 | BIG | Moneymaker | Fresh Market | *Solanum lycopersicum* | - |
| 111 | BIG |  | Processing tomato | *Solanum lycopersicum* | - |
| 113 | BIG | Hacienda Calera | Landrace/Latin American cultivar | *Solanum lycopersicum* | LA0113 |
| 114 | BIG | Pearson | Vintage Processing | *Solanum lycopersicum* | LA0012 |
| 128 | BIG |  |  | *Solanum lycopersicum* | LA3130 |
| 132 | BIG | T-5 | Modern Fresh Market | *Solanum lycopersicum* | LA2399 |
| 149 | BIG | Tegucigalpa | Landrace/Latin American cultivar | *Solanum lycopersicum* | LA0147 |
| 166 | BIG | Tarapoto |  | *Solanum lycopersicum* | LA2283 |
| 176 | BIG |  |  | *Solanum lycopersicum* | LA3238 |
| 180 | BIG | Hot set | cultivar | *Solanum lycopersicum* | LA3320 |
| 183 | BIG | Prince Borghese | Vintage Fresh Market | *Solanum lycopersicum* | LA0089 |
| 208 | BIG |  | Processing tomato | *Solanum lycopersicum* | - |
| 252 | BIG | Early Santa Clara | Vintage Processing | *Solanum lycopersicum* | LA0517 |
|  |  |  |  |  |  |

Table S6. Annual precipitation and its proportion of different tomato classes. 1/4: the quarter digit of the precipitation, 1/2: the median, 3/4: the three quarters; PIM: *S. pimpinellifolium*, Xinjiang: Xinjiang tomato, Winter: Winter tomato.

| **Class** | **1/4** | **1/2** | **3/4** | **Arid** | **Semi-arid** | **Semi-humid** | **Humid** |
| --- | --- | --- | --- | --- | --- | --- | --- |
| PIM | 56 | 255.5 | 1112 | 43.90% | 15.85% | 12.20% | 28.05% |
| Xinjiang | 125.5 | 150 | 183 | 82.76% | 17.24% | / | / |
| Winter | 1345 | 1553.5 | 1711 | / | / | 4.55% | 95.45% |

Table S7. Aridity index and its proportion of different tomato classes. 1/4: the quarter digit of the precipitation, 1/2: the median, 3/4: the three quarters; PIM: *S. pimpinellifolium*, Xinjiang: Xinjiang tomato, Winter: Winter tomato.

| **Class** | **1/4** | **1/2** | **3/4** | **Arid** | **Semi-arid** | **Dry sub-humid** | **Humid** |
| --- | --- | --- | --- | --- | --- | --- | --- |
| PIM | 362 | 1777.5 | 8159 | 53.05% | 15.85% | 2.44% | 28.66% |
| Xinjiang | 783.5 | 1008 | 1278.5 | 89.66% | 7.69% | 3.45% | / |
| Winter | 8847.5 | 11001.5 | 11726 | / | 2.27% | 4.55% | 93.18% |

Table S8. Available water storage capacity in mm/m of the soil unit of different tomato classes. 1/4: the quarter digit of the precipitation, 1/2: the median, 3/4: the three quarters; PIM: *S. pimpinellifolium*, Xinjiang: Xinjiang tomato, Winter: Winter tomato.

| **Class** | **1/4** | **1/2** | **3/4** |
| --- | --- | --- | --- |
| PIM | 50 | 100 | 150 |
| Xinjiang | 150 | 150 | 150 |
| Winter | 150 | 150 | 150 |

Table S9. The average AUC values obtained in the models for both current and future periods (n = 10).

| **Climatic data set**  **Periods** | **19 bio** | **summer** | **winter** |
| --- | --- | --- | --- |
| current | 0.897 | 0.879 | 0.871 |
| 2021-2040 | \ | 0.879 | 0.871 |
| 2041-2060 | \ | 0.879 | 0.871 |
| 2061-2080 | \ | 0.879 | 0.871 |
| 2081-2100 | \ | 0.879 | 0.871 |

Table S10. Highly suitable range and the most suitable values of Annual Precipitation (bio 12), Precipitation of Driest Quarter (bio 17), Isothermality (BIO2/BIO7) (* 100) (bio 3), Precipitation of Driest Month (bio 14), Precipitation of Wettest Quarter (bio 16), Precipitation of Coldest Quarter (bio 19).

| **Climatic data** | **Highly suitable range** | **The most suitable value** |
| --- | --- | --- |
| Annual Precipitation (bio 12)(mm) | 6.34-572.53 | 37.07 |
| Precipitation of Driest Quarter (bio 17)(mm) | 0.76-10.29 | 0.99 |
| Isothermality (BIO2/BIO7) (* 100) (bio 3)  Precipitation of Driest Month (bio 14) (mm)  Precipitation of Wettest Quarter (bio 16) (mm)  Precipitation of Coldest Quarter (bio 19) (mm) | 35.1-76.93  0.23-4.77  1.99-308.37  1.03-52.84 | 35.1  0.23  33.11  1.03 |

Table S11. The RI (Recovery Index) values of domesticated tomato and *S. pimpinellifolium*.

| PIM_Number | RI | BIG_Number | RI |
| --- | --- | --- | --- |
| **14** | 35% | **2** | 93% |
| **15** | 100% | **3** | 58% |
| **18** | 75% | **4** | 68% |
| **20** | 69% | **9** | 97% |
| **21** | 78% | **45** | 93% |
| **23** | 83% | **60** | 68% |
| **24** | 86% | **77** | 50% |
| **25** | 53% | **84** | 94% |
| **67** | 55% | **111** | 93% |
| **81** | 100% | **113** | 90% |
| **127** | 98% | **114** | 96% |
| **135** | 98% | **128** | 50% |
| **238** | 50% | **132** | 88% |
| **265** | 98% | **149** | 81% |
| **277** | 58% | **166** | 56% |
| **278** | 88% | **176** | 75% |
| **279** | 75% | **180** | 67% |
| **280** | 68% | **183** | 63% |
| **281** | 94% | **208** | 94% |
| **289** | 92% | **252** | 67% |
