# Supplementary material for: Prediction of suitable regions of wild tomato provides insights on domesticated tomato cultivation in China
Source: BMC Plant Biol. 2024 Jul 22;24:693. doi: 10.1186/s12870-024-05410-z (PMC11265077; doi:10.1186/s12870-024-05410-z)
Supplement: Supplementary file 2 — Supplementary Material 2. [file 12870_2024_5410_MOESM2_ESM.docx]

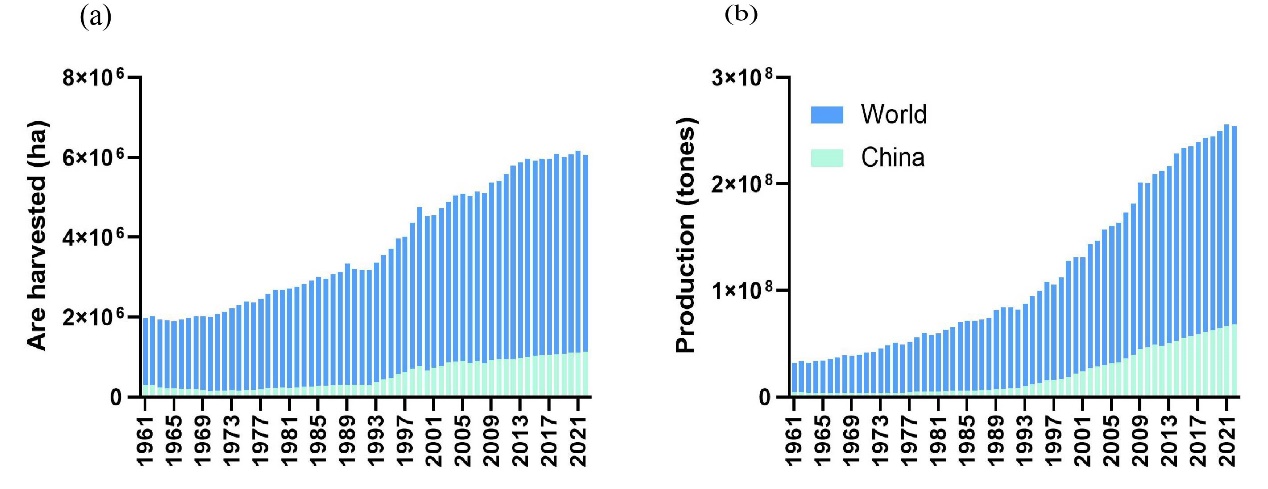


Fig. S1. Area harvested (a) and Production (b) of domesticated tomato in China and worldwide from 1961-2022.


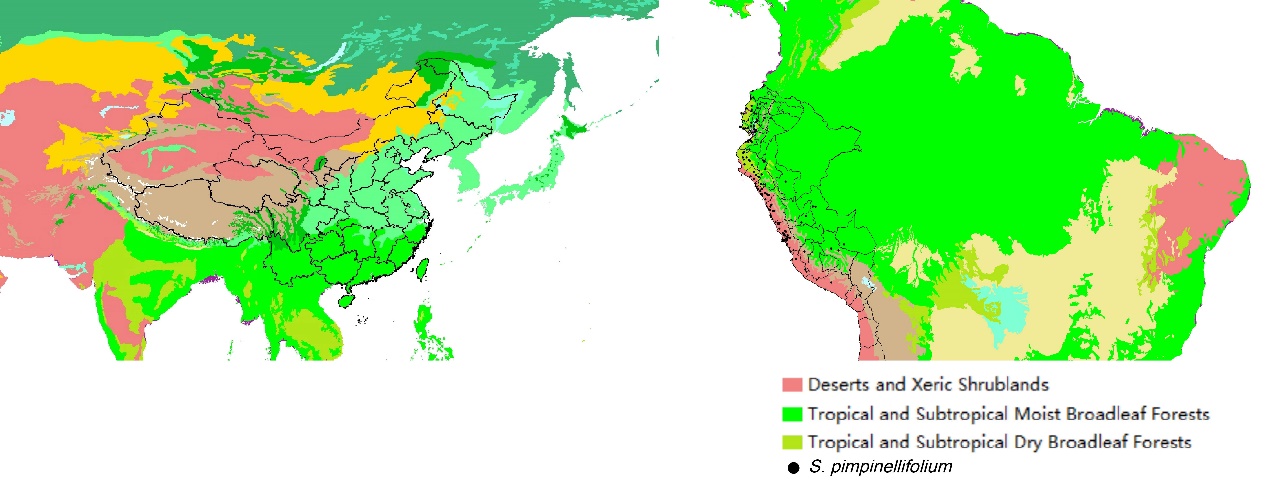


Fig. S2. Biomes map of the native range of *S. pimpinellifollium* in South American and biomes map of China (ArcGIS v10.8, ESRI, 2020.)[1, 2].


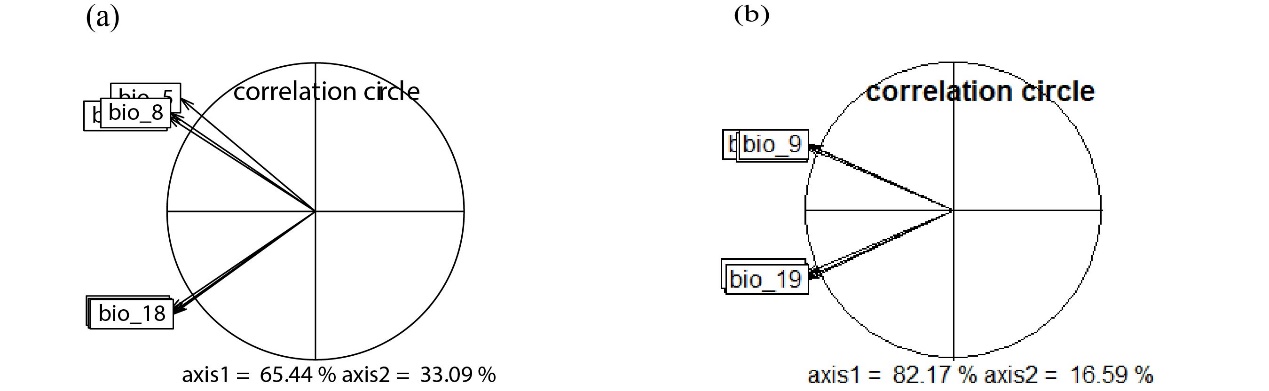


Fig. S3. The contribution of the climatic variables on two axes of the PCA. (a) summer production seasons, (b) winter production seasons.


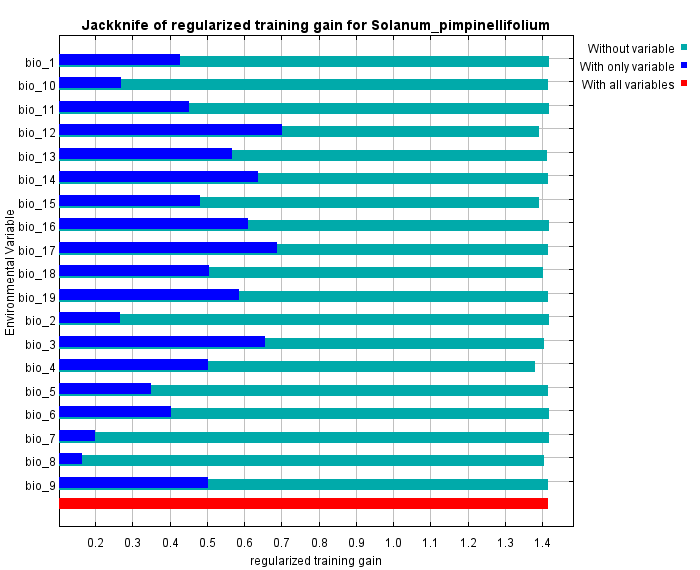


Fig. S4. Results of Jackknife test of MaxEnt modelling using 19 bioclimatic variables.


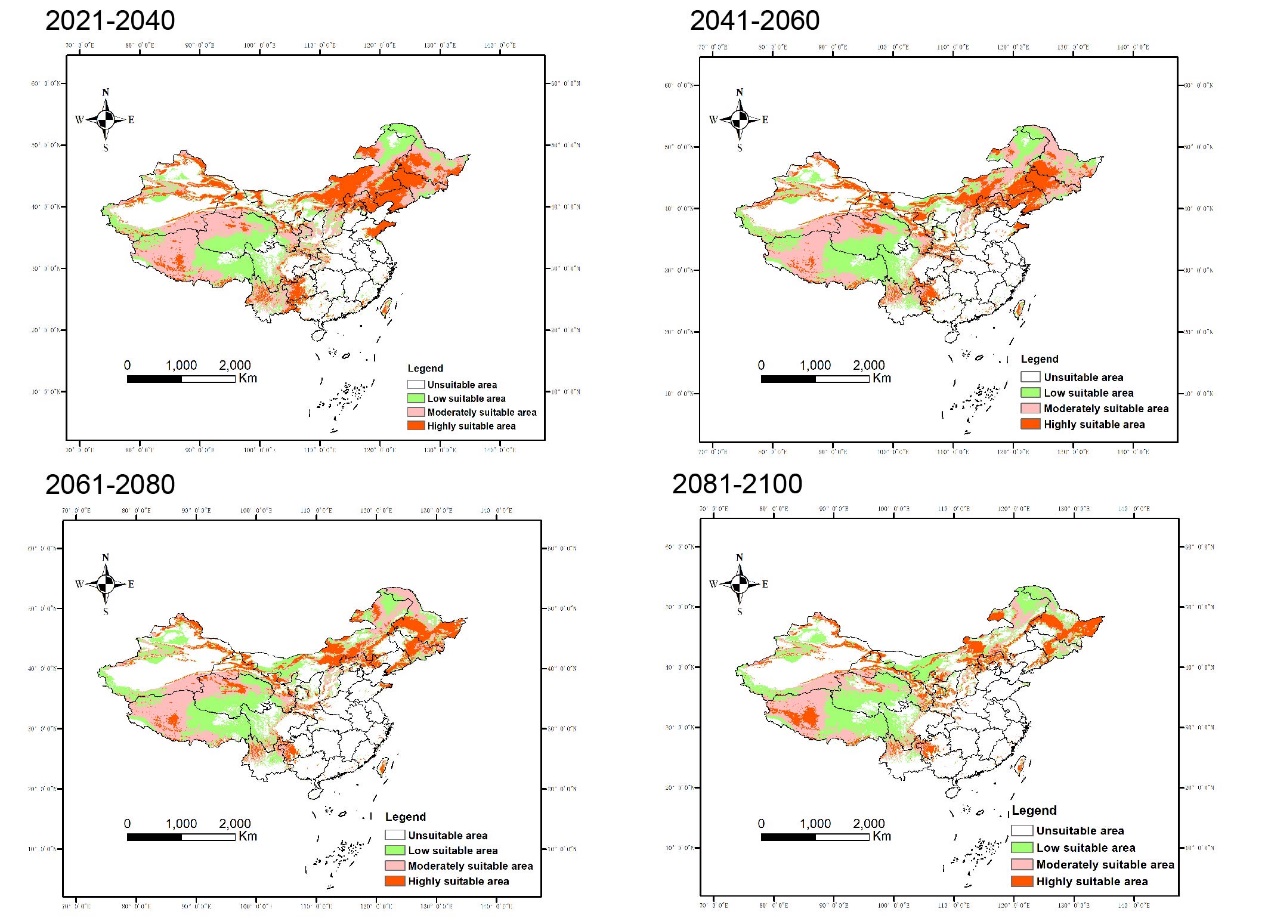


Fig. S5. The predicted land suitability for *S. pimpinellifolium* in China by using summer variables (bio 5, bio 8, bio 10, bio 13, bio 16, bio 18) under future climate scenarios (2021-2040, 2041-2060, 2061-2080, 2081-2100).


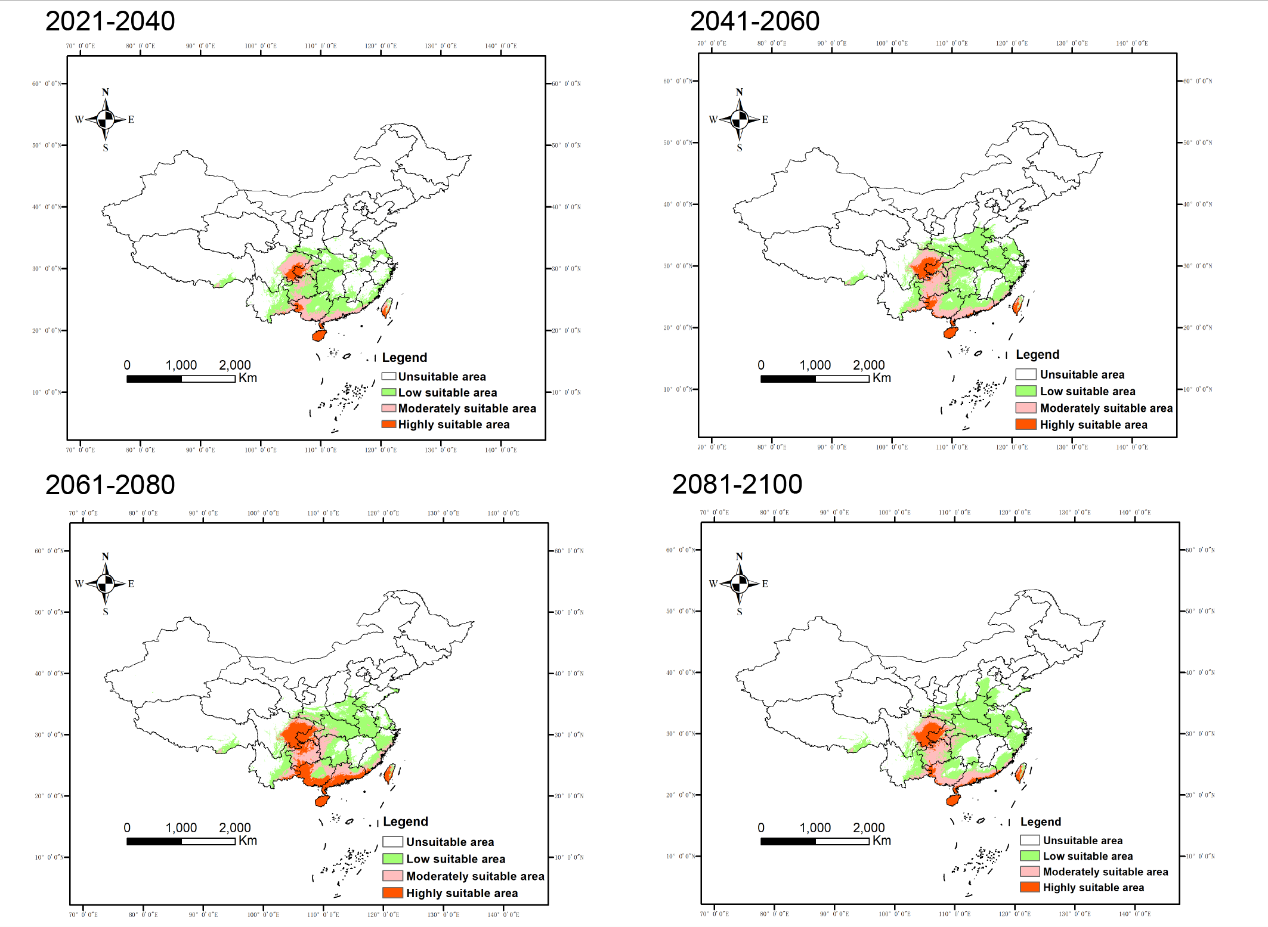


Fig. S6. The predicted land suitability for *S. pimpinellifolium* in China by using winter variables (bio 6, bio 9, bio 11, bio 14, bio 17, bio 19) under future climate scenarios (2021-2040, 2041-2060, 2061-2080, 2081-2100).


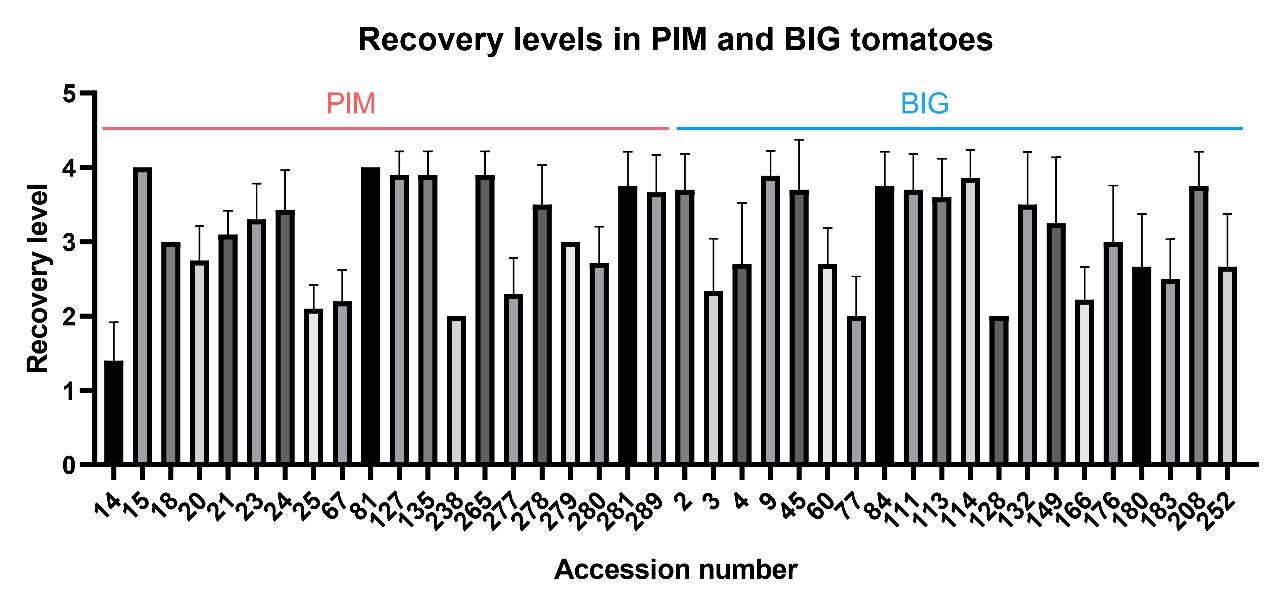


Fig. S7. The recovery levels in the PIM and BIG tomatoes.

**References**

1. Olson DM, Dinerstein E, Wikramanayake ED, Burgess ND, Powell GV, Underwood EC, D'amico JA, Itoua I, Strand HE, Morrison JC: **Terrestrial Ecoregions of the World: A New Map of Life on Earth: A new global map of terrestrial ecoregions provides an innovative tool for conserving biodiversity**. *BioScience* 2001, **51**(11):933-938.

2. Ramírez-Ojeda G, Peralta IE, Rodríguez-Guzmán E, Chávez-Servia JL, Sahagún-Castellanos J, Rodríguez-Pérez JE: **Climatic Diversity and Ecological Descriptors of Wild Tomato Species (Solanum sect. Lycopersicon) and Close Related Species (Solanum sect. Juglandifolia y sect. Lycopersicoides) in Latin America**. *Plants* 2021, **10**(5):855.
